# Comparable emotional dynamics in women with ADHD and borderline personality disorder

**DOI:** 10.1186/s40479-021-00144-y

**Published:** 2021-02-12

**Authors:** Talar R. Moukhtarian, Iris Reinhard, Paul Moran, Celine Ryckaert, Caroline Skirrow, Ulrich Ebner-Priemer, Philip Asherson

**Affiliations:** 1 King’s College London, Social, Genetic and Developmental Psychiatry Centre, Institute of Psychiatry, Psychology and Neuroscience, London, UK; 2 Central Institute of Mental Health, Division of Biostatistics, Medical Faculty Mannheim/Heidelberg University, Mannheim, Germany; 3 Centre for Academic Mental Health, School of Social & Community Medicine, University of Bristol, Bristol, UK; 4 Cambridge Cognition, Cambridge, UK; 5 School of Psychological Science, University of Bristol, Bristol, UK; 6 Mental m-health lab, Institute of Sports and Sports Science, Karlsruhe Institute of Technology (KIT), Karlsruhe, Germany; 7 Department of Psychiatry and Psychotherapy, Central Institute of Mental Health, Medical Faculty Mannheim, Heidelberg University, Heidelberg, Germany

**Keywords:** Emotional dysregulation, Attention-deficit/hyperactivity disorder, Borderline personality disorder, Experience sampling method, Transdiagnostic

## Abstract

**Background:**

Emotional dysregulation (ED) is a core diagnostic symptom in borderline personality disorder (BPD) and an associated feature of attention-deficit/hyperactivity disorder (ADHD). We aimed to investigate differences in dynamical indices of ED in daily life in ADHD and BPD.

**Methods:**

We used experience sampling method (ESM) and multilevel modelling to assess momentary changes in reports of affective symptoms, and retrospective questionnaire measures of ED in a sample of 98 adult females with ADHD, BPD, comorbid ADHD+BPD and healthy controls.

**Results:**

We found marked differences between the clinical groups and healthy controls. However, the ESM assessments did not show differences in the intensity of feeling *angry* and *irritable*, and the instability of feeling *sad*, *irritable* and *angry,* findings paralleled by data from retrospective questionnaires. The heightened intensity in negative emotions in the clinical groups compared to controls was only partially explained by bad events at the time of reporting negative emotions, suggesting both reactive and endogenous influences on ED in both ADHD and BPD.

**Conclusions:**

This study supports the view that ED is a valuable trans-diagnostic aspect of psychopathology in both ADHD and BPD, with similar levels of intensity and instability. These findings suggest that the presence or severity of ED should not be used in clinical practice to distinguish between the two disorders.

**Supplementary Information:**

The online version contains supplementary material available at 10.1186/s40479-021-00144-y.

## Background

Differentiating attention-deficit/hyperactivity disorder (ADHD) from borderline personality disorder (BPD) is clinically important to facilitate the correct management of both conditions. Yet, some overlapping symptoms, notably emotional dysregulation (ED), can make differentiation of the conditions challenging [1, 2]. According to the DSM-5, ED reflects a core symptom domain in the diagnostic classification of BPD, whereas in ADHD it is recognised as an associated feature supporting the diagnosis [3]. A scoping review by Moukhtarian, Mintah [2] describes in detail the similarities and differences of ED in ADHD and BPD.

Research shows that ED, characterised by problems with temper control, emotional over-reactivity, and mood lability, is present in 72–90% of adults with ADHD [4]. ED, independently of core ADHD symptoms, predicts impairments in social, educational and occupational domains [5–7]. ED also shows moderate reduction following treatment with stimulants or atomoxetine [8, 9].

In BPD, ED is characterised by severe feelings of heightened and unstable reactivity of mood (DSM-5 criterion six) and difficulty controlling anger (DSM-5 criterion eight) [3], substantially impacting in an enduring way on quality of life and psychosocial functioning [10].

Experience sampling methods (ESM), also referred to as ecological momentary assessment (EMA) [11] can be used to investigate a dynamic and time-varying phenomenon such as ED [12]. ESM uses repeated real-time assessments of affective states and physiological processes in their natural environment [11, 13], capturing stability and change over time [14, 15], minimising retrospective and heuristic biases [16], and providing longitudinal data with high ecological validity [12].

So far, only one ESM study has investigated ED in adults with ADHD [17]. In an all-male non-comorbid sample, increased instability and intensity of negative emotions were self-reported in ADHD compared to controls, but no differences in the intensity and instability of positive emotions. Compared to controls, ESM studies in BPD [16, 18–21] have found heightened affective instability of positive and negative emotions, and greater intensity of negative emotions, with no group differences in the intensity of positive emotions.

Several other studies investigated differences in emotion intensity and instability in BPD compared to other clinical groups. Compared to participants with depression, those with BPD reported a greater long-term (between-day) and short-term (within-day) instability of negative emotions [15, 22–24], but a comparable intensity of positive and negative emotions [22]. In a sub-sample of the same study, BPD with post-traumatic stress disorder (PTSD) showed greater instability of negative emotions compared to the BPD only and depression groups [25]. However, similar levels of affective instability were reported by all diagnostic groups in a direct comparison of BPD, PTSD, and bulimia nervosa, although affective instability was still heightened in these clinical groups in comparison with healthy controls [26, 27].

Overall, these ESM studies support the notion that affective instability is not disorder-specific. And while results are consistent with overlapping symptoms of ED in ADHD and BPD as shown using retrospective questionnaire measures [28–30], to date no studies have used ESM to evaluate the overlapping dynamic construct of ED between ADHD and BPD. It remains unclear whether the type of ED seen in ADHD is distinct from that seen in BPD [2].

ADHD and BPD have both been associated with greater adversity and acute stressful situations in everyday life [31, 32]. This raises the question of whether ED might reflect a response to more frequent adverse situations. Two studies in BPD using an event-contingent sampling strategy, querying about characteristics of social interactions such as time, environment, interaction partners [33, 34], reported no differences in variability of negative emotions between BPD and control groups. Skirrow and colleagues found that participants with ADHD showed greater reactivity of negative emotions, such as anger, to reported bad life events than control participants, albeit not using an event-contingent sampling [17]. However, increased intensity and instability of negative emotions were not entirely accounted for by reported bad events. ED in ADHD and BPD could potentially be differentiated by response to different types of adverse events. Greater sensitivity and heightened reactivity involving interpersonal events/stressors have been shown in BPD [34], in addition to the classification of disturbed interpersonal relationships as a diagnostic symptom in BPD [3].

We conducted a direct comparison of ADHD with BPD using an ESM approach. The study investigated the dynamics of positive and negative emotions and explored the occurrence and impact on intensity of negative emotions of self-reported adverse events (*bad social* events; involving other people or social situations, and *bad functional* events; involving practical and everyday life tasks), rated eight times daily over 5 days, in adult females with ADHD only, BPD only, comorbid ADHD+BPD, and controls. We hypothesised that in line with differences in diagnostic criteria for ADHD and BPD, participants with BPD would show heightened intensity and instability of positive and negative emotions compared to participants with ADHD. And consistent with criterion two of the DSM-5; “pattern of unstable and intense interpersonal relationships” in BPD, we further hypothesised that *bad social* events will be a stronger predictor of negative mood reactivity in BPD compared to ADHD, potentially setting apart the two conditions.

## Methods

### Sample

From a sample of 114 eligible participants, 105 provided ESM data. Seven were excluded due to low ESM compliance (details in "Pre-processing of ESM data"), with a final sample of 98 participants used in this study. Controls, screened with the Barkley Adult ADHD Rating Scale [35] and the Structured Clinical Interview for DSM-IV axis II disorders- BPD items [36], and not meeting criteria for ADHD or BPD respectively, were recruited through advertisements in King’s College London, volunteer databases, and within the local community. Clinical cases were recruited from ADHD and borderline personality specialist clinics in the South and North London and Midland regions of England. Members of the clinical care teams identified potentially eligible participants (i.e. based on clinical judgment of suspected ADHD in the BPD clinics, or vice versa, and study inclusion/exclusion criteria) and referred them to the research team. Clinician diagnoses were based on DSM-5 criteria for ADHD and BPD [3], and validated for research by members of the research team using the Diagnostic Interview for ADHD in Adults (DIVA) [37] and the Zanarini rating scale for Borderline Personality Disorder (ZAN-BPD) [38] to maintain reliability and consistency of diagnosis across the whole sample (see section 1 in the supplementary materials for further details of the clinical research diagnosis). Co-morbidities were excluded using a checklist of common mental health conditions by screening clinical case records. Exclusion criteria for the clinical and control groups were- male gender; history of bipolar I and II, recurrent depressive episodes, and schizophrenia; current Axis I disorders; head injury or neurological conditions; IQ < 70; and current treatment with mood stabilisers, anti-psychotics and atomoxetine. Participants on stimulant medication for ADHD were asked to come off this medication 48 h before the baseline assessment and the following 5 days during the experience sampling week. Due to the frequent drug and alcohol use in ADHD [39–41] and BPD [42, 43], we excluded individuals with substance dependence syndromes (based on an official substance dependence diagnosis or clinical notes of clinicians), but included individuals who reported harmful and excessive use of alcohol and drugs (see section 2 in the supplementary materials for screening measures and sensitivity analyses).

### Measures

#### Symptom measures

ED was assessed using one self-rated questionnaire and one investigator-rated interview scale; the Affective Lability Scale- Short form (ALS-SF) [44] and the Wender-Reimherr Adult Attention Deficit Disorder Scale- Emotion dysregulation subscale (WRAADDS-EDS) [45] respectively. Co-occurring symptoms of depression and anxiety were measured by subscales of the Brief Symptom Inventory (BSI) [46]. Intellectual function (IQ) was estimated using two subtests (vocabulary and matrix reasoning) of the Wechsler Abbreviated Scale of Intelligence- Second edition [47] (see section 3 in supplementary materials for details of measures used).

#### Experience sampling of emotions

ESM was carried out using a pseudorandomised fixed schedule of rating periods, eight times daily, across five consecutive days, according to established procedures [17]. We used an iOS app called MoodMapper, uploaded onto Apple iPods with all other functions disabled. Signals for the onset of each monitoring period were provided by ‘Vibralite 12’ wristwatches giving silent vibration signals that were synchronised with the iPods. Participants were instructed to complete each rating basing their responses on the time-period just before the signal. Start and end times were the same each day, starting at 9:30 in the morning, and finishing at about 7:30 in the evening, with each monitoring instance lasting no more than 2 min.

MoodMapper employed a total of five continuous analogue scale questions on negative and positive emotions with ratings ranging from 0 (not at all) to 100 (extremely), and one categorical multiple-choice question (see Table 1).

**Table 1:** Moodmapper mood items with descriptions and scoring

Items	Description	Scoring
Item1- Happy	How **happy** do you feel now?	0 ………………...100
Item2- Excited	How **excited** do you feel now?	0 ………………...100
Item3- Sad	How **sad** do you feel now?	0 ………………...100
Item4- Irritable	How **irritable** do you feel now?	0 ………………...100
Item5- Angry	How **angry** do you feel now?	0 ………………...100
Item6	Did any bad thing happen to you in the past hour?	1. No 2. Argument^a^ 3. Lost something^b^ 4. Late/missed something I wanted^b^ 5. Told off^a^ 6. Punished^a^ 7. Hurt/accident/pain^b^ 8. Annoyed by someone^a^ 9. Bullied^a^ 10. Failed something^b^ 11. Need to do something I dislike^b^ 12. Other

^a^ Items grouped in *bad social events* category; ^b^ Items grouped in *bad functional events* category

### Statistics

#### Pre-processing of ESM data

To reduce self-selection bias of monitoring instances, all reports not completed within 16-min of the signal were excluded from analyses [15, 17]. Compliance rates were computed as the proportion of responses completed within the 16-min window (maximum 40). In line with previous studies [17, 48], participants with less than 40% compliance were excluded from analyses (*n* = 7).

To obtain a measure of emotion instability, we calculated squared successive differences (SSD) for each continuous item: the squared value of the difference between successive responses (t_i_-t_i-1_)^2^ [18]. SSD is a robust measure for systematic time trends in time series data [23, 49] evaluating change from one rating to the next [22], incorporating amplitude and frequency of change, and temporal dependency of ratings [23]. (See section 4 in the supplementary materials for further details on the pre-processing of ESM data).

Finally, multiple choice answers for the bad events question (item 6 in Table 1) were grouped into two categories; (1) *bad social events*: argument, told off, punished, annoyed by someone, bullied; which were events involving other people, and (2) *bad functional events*: lost something, late/missed something I wanted, hurt/accident/pain, failed something, need to do something I dislike; which were non-social events relating to everyday life situations and involving the subject only. The total number of events reported for each category across the whole rating period per participant was then calculated and used as variables in subsequent exploratory analyses.

#### Data analyses

Analyses were carried out in SAS university edition- virtualbox and SPSS 26. The significance level α was held at .05 (two-tailed). Mean ratings were computed for each questionnaire-based self-report measure and compared between groups. Normality of data was assessed by examining histograms and QQ plots, and with the Shapiro-Wilk statistic. Parametric and non-parametric Kruskal-Wallis tests were used, as appropriate. For ESM data, multilevel models were used to account for correlated observations nested within individuals, which also perform well with missing data [13, 23].

In the multilevel models, adjustments for multiple testing contrast tests were made by applying Bonferroni and Bonferroni-Holm corrections. We further made Bonferroni correction across the five ESM items and used an adjusted *p* = .01 for the multilevel analyses. Main findings reported in this paper are focused on the ESM analyses, we therefore did not correct for multiple testing for the analyses of questionnaire-based measures.

We investigated differences across the four groups by contrasts, evaluating (1) intensity of emotions using raw data and (2) instability of emotions using SSDs. Normally distributed data were analysed with a linear multilevel model; a linear mixed model with a random intercept (SAS procedure GLIMMIX). As an example, we present the below model single equation representation we used to calculate intensity of emotions with a main effect of group.

$$ {Y}_{\mathrm{ij}}={\beta}_{00}+{\beta}_{10}\ast ADHD{\left( yes/ no\right)}_{\mathrm{j}}+{\beta}_{20}\ast BPD{\left( yes/ no\right)}_{\mathrm{j}}+{\beta}_{30}\ast ADHD\ and\  BPD{\left( yes/ no\right)}_{\mathrm{j}}+{\mathrm{u}}_{0\mathrm{j}}+{\varepsilon}_{\mathrm{ij}} $$

Here, *Y*_*ij*_ represents the level of emotion intensity at time *i* for person *j*. The *β* coefficients represent the intercept and the fixed main effects for group, while the *u*_*0j*_ denote random intercepts for person *j* and the *ε*_*ij*_ the residuals at level 1.

SSDs follow a χ^2^ distribution, which is a special case of the gamma distribution and were analysed with generalised multilevel models with gamma distributions and log links (SAS procedure GLIMMIX), which relies on linearization and Taylor series techniques, to construct Wald-type test statistics and confidence intervals to estimate these models [50]. For further details on the equations we used for the gamma models, please refer to the online supplement appendix S3 by Santangelo and colleagues [50].

Regarding self-reported *bad social* and *bad functional* events, we run a set of exploratory analyses. First, we investigated differences in the frequency of reported events in the groups. Second, multilevel models were run to investigate the relative contribution of bad events in the intensity of negative emotions across the whole sample and per group, and finally, additional models were run with a group (ADHD, BPD, comorbid ADHD+BPD and control) by bad events interaction to investigate potential group differences in negative emotion reactivity to bad events.

Given the significant co-occurrence of depression and anxiety in both ADHD [51] and BPD [52] populations, we also explored potential confounding effects of depression and anxiety symptoms on ED, by incorporating depressive and anxious symptoms as main effects within models.

## Results

### Sample characteristics and compliance

Group demographics and post-hoc comparisons are documented in Table 2. The sample consisted of 98 females between the ages of 18–65 years (M_age_ = 33.4, SD = 11.4): 28 with ADHD only, 19 with BPD only, 22 with comorbid ADHD+BPD, and 29 controls. The groups significantly differed on age, X^2^(3) = 14.53, *p* = .002, and IQ, F (3,93) = 4.6, *p* = .005. Both age and IQ were initially controlled for in the analyses of retrospective report measures and ESM data but did not have significant effects in the models. We therefore report models unadjusted for age and IQ. There were no group differences in ESM compliance rates (X^2^(3) = .12, *p* = .989; M = 74.8%, SD = 14.9).

**Table 2:** Descriptive statistics and pairwise comparisons of measures

	Control^**1**^ (***n*** = 29)	ADHD^**2**^ (***n*** = 28)	BPD^**3**^ (***n*** = 19)	ADHD + BPD^**4**^ (***n*** = 22^≠^)	Post-hoc
**Measures**	***Mean (SD)***	***Mean (SD)***	***Mean (SD)***	***Mean (SD)***	
***Age***	27.1 (5.2)	38.2 (11.7)	35.4 (11.4)	33.8 (13.8)	1 < 2**
***IQ***	107.2 (9.2)	106.5 (14.2)	97 (13.8)	97.7 (12.4)	1 > 3*
***Compliance rate in %***	75.6 (14.6)	74.5 (14.1)	73.8 (16.7)	74.8 (15.7)	–
***ALS-SF***	7.4 (7.3)	29.4 (13.7)	33.8 (10.4)	37.9 (10.5)	1 < 2,3,4***
***WRAADDS-EDS***	2.9 (2.5)	13.6 (3.4)	14.9 (2.6)	17.3 (2.4)	1 < 2,3,4***; 2 < 4**
**Total ZAN-BPD**	0.7(1.3)	6.8(4)	20.6(4.2)	23.8(6.3)	1 ≤ 2,3,4** 2 < 3,4***
**Total current DIVA**	1(1.3)	13.5(2.6)	5.5(4)	12.3(2.9)	1 ≤ 2,3,4* 3 < 2***; 3 < 4**
**BSI_Depression**	1.8 (2.2)	5.3 (4.8)	14 (6.3)	15.9 (5.7)	1 < 3,4***; 2 < 3, 4***
**BSI_Anxiety**	1.3 (1.6)	7.2 (4.4)	12.7 (4.8)	15.9 (5.4)	1 < 2,3,4***; 2 < 4***
**Sum of bad social**	2.2 (2.6)	4.4 (3.2)	6.9 (6.2)	5.5 (4.6)	1 < 2*; 1 < 3,4**
**Sum of bad functional**	3 (3.5)	9.1 (8.3)	6 (4.8)	6.4 (7.4)	1 < 2**

Key: SD: Standard Deviation, IQ: Intelligent Quotient, ALS-SF; Affective Lability Scale- Short Form, WRAADDS-EDS; Wender-Reimherr Adult Attention Deficit Disorder Scale- Emotion Dysregulation Subscale, ZAN-BPD; Zanarini rating scale for Borderline Personality Disorder, DIVA; Diagnostic Interview for ADHD in Adults, BSI; Brief Symptom Inventory; Sum of bad social/Sum of bad functional: average number of bad events over the 5-day experience sampling period; 1: Control group; 2: ADHD only group; 3: BPD only group; 4: Comorbid ADHD+BPD group

### Retrospectively measured ADHD and BPD symptoms, emotional dysregulation, depression and anxiety

Descriptive statistics and group comparisons of the measures are listed in Table 2.

Kruskal-Wallis tests revealed significant group differences in ADHD and BPD symptoms measured by retrospective report by the DIVA and ZAN-BPD respectively (X^2^(3)= 82.41, *p* < .001 for the ZAN-BPD, and X^2^(3)= 72.23, *p* < .001 for the DIVA). Post-hoc analyses indicated that all three clinical groups had significantly more BPD symptoms than the control group (*ps* < .01). Additionally, the ADHD group had significantly less BPD symptoms than the BPD and comorbid ADHD+BPD groups (*p* < .001), whereas no significant differences were seen between the BPD and comorbid ADHD+BPD groups (*p* = 1). Post-hoc analyses also indicated that the three clinical groups had elevated current ADHD symptoms compared to controls (*ps* < .05). The BPD group had significantly less ADHD symptoms compared to both the ADHD (*p* < .001) and comorbid ADHD+BPD (*p* < .01) groups, who showed no differences between each other on the measure (*p* = 1).

Kruskal-Wallis tests revealed significant group differences in ED measured by retrospective report (X^2^(3) = 52.21, *p* < .001 for the ALS-SF, and X^2^(3) = 68.34, *p* < .001 for the WRAADDS-EDS). Post-hoc tests showed significantly elevated ED in all clinical groups compared to controls on both scales (*p* < .001). The three clinical groups did not differ on the ALS (*ps* ≥ .41). Regarding the WRAADDS-EDS, the ADHD+BPD group reported significantly elevated ratings compared to the ADHD group (*p* = .005), and comparisons between all other clinical groups were non-significant (*ps ≥* .44).

There were significant group differences in depression, X^2^(3) = 61.85, *p* < .001 and anxiety, X^2^(3) = 66.59, *p* < .001. Post-hoc tests showed significantly elevated depression and anxiety in the clinical groups compared to controls (*p* < .001), except non-significant differences in depression between ADHD and controls (*p* = .08). Additionally, the ADHD group showed less depression and anxiety compared to comorbid ADHD+BPD (*p* < .001), and less depression compared to BPD (*p* = .001). No differences were seen between the ADHD and BPD groups on anxiety (*p* = .07), and between the BPD and ADHD+BPD groups on both subscales (*p* = 1).

### Real-time emotional changes

#### Intensity

Multilevel models revealed a significant main effect of group on the intensity of all positive and negative emotion items (*Happy*: F(3, 94.07) = 11.96, *p* < .001; *Excited*: F(3, 94.06) = 4.44, *p* = .006; *Sad*: F(3, 94.17) = 15.04, *p* < .001; *Irritable*: F(3, 93.66) = 19.61, *p* < .001; *Angry*: F(3, 94.21) = 9.47, *p* < .001).

Case-control post-hoc comparisons (see Table 3) showed a significantly higher intensity of *happy*, and a significantly lower intensity of all negative emotion items (*sad*, *irritable* and *angry*) in the control group compared to the BPD and ADHD+BPD groups *(ps* ≤ .0002). The control group also reported significantly elevated intensity of *excited* compared to the BPD group (*p* = .011) only. Compared to controls, the ADHD group reported heightened intensity of *irritable* (*p* < .0001), but no differences were seen between these two groups on the intensity of *sad* (*p* = .046) and *angry* (*p* = .097), and positive emotion items (*happy*, *p* = .430; *excited*, *p* = .816). All significant findings were robust to Bonferroni correction (adjusted *p* = .01).

**Table 3:** Estimated means^a^, standard errors, and group comparisons from intensity models

Intensity of	Estimated Mean (Standard Error)				Post-hoc
	Control^1^	ADHD^2^	BPD^3^	ADHD+BPD^4^	
Happy	56.83 (2.76)	51.91 (2.81)	36.16 (3.41)	36.92 (3.17)	1 > 3,4***; 2 > 3,4**
Excited	42.24 (3.12)	38.53 (3.18)	26.36 (3.86)	30.20 (3.59)	1 > 3**
Sad	15.88 (3.25)	26.60 (3.31)	45.42 (4.02)	42.12 (3.73)	1 < 3,4***; 2 < 3,4**
Irritable	17.51 (2.87)	36.03 (2.93)	44.75 (3.56)	47.44 (3.30)	1 < 2,3,4***; 2 < 4* (*p* = .03)
Angry	12.53 (2.91)	20.83 (2.96)	32.51 (3.59)	32.39 (3.34)	1 < 3,4***; 2 < 3,4* (*p* = .04)

Key: *p ≤ .05, **p ≤ .01, ***p ≤ .001; Bold characters indicate findings NOT withstanding Bonferroni correction at *p* = .01; 1: Control group; 2: ADHD only group; 3: BPD only group; 4: Comorbid ADHD+BPD group

^a^ Means of squared successive difference of items used in the intensity models

Post-hoc comparisons in the clinical groups showed no differences in the intensity of all positive and negative emotion items (*happy*, *excited*, *sad*, *irritable*, and *angry*) between the BPD and comorbid ADHD+BPD groups (*ps* ≥ .54). Participants with ADHD showed significantly higher intensity of *happy* (*p* ≤ .01), and a significantly lower intensity of *sad* (*p* ≤ .01) and *angry* (*p* < .05) compared to the BPD and comorbid ADHD+BPD groups, and a significantly lower intensity of *irritable* (*p* = .034) compared to the comorbid ADHD+BPD group. The clinical groups did not differ in the intensity of *excited* (*ps* ≥ .07). Differences between the clinical groups on intensity of *angry* and *irritable* were no longer significant after correction for multiple testing (adjusted *p* = .01).

In models adjusted for anxiety and depression, main effect of group dissipated for all positive and negative mood items (*p* ≥ .38), except *irritable* (F(3, 91.81) = 2.87*, p* = .04), whereby the ADHD group reported significantly elevated intensity of *irritable* compared to the control group. However, this finding was no longer significant after correction for multiple testing (adjusted *p* = .01) (See section 5 in the supplementary materials and Table S1 for adjusted estimated means of the intensity models).

#### Instability

Multilevel models revealed a main effect of group on the instability of all items (*Happy*: F(3, 91.21) = 2.88, *p* = .04; *Sad*: F(3, 92.84) = 7.67, *p* < .001; *Irritable*: F(3, 92.5) = 7.57, *p* < .001; *Angry*: F(3, 94.65) = 9.06 *p* < .001), except *excited* (F(3, 95.18) = 1.46, *p* = .23). Only instability models for the negative emotion items remained significant after correcting for multiple testing (adjusted *p* = .01).

Post-hoc comparisons (see Table 4) showed a significantly heightened instability of all negative emotion items (*sad*, *irritable*, *angry*) in the three clinical groups compared to controls (*p* ≤ .01). There were no differences in the instability of *sad*, *irritable* and *angry* between the three clinical groups (*ps* ≥ .16).

**Table 4:** Estimated means^a^, standard errors, and group comparisons from instability models

Instability of	Estimated Mean (Standard Error)				Post-hoc
	Control^1^	ADHD^2^	BPD^3^	ADHD+BPD^4^	
Happy	250.55 (39.08)	371.68 (59.15)	342.10 (66.03)	498.64 (89.27)	1< 4* (*p* = .03)
Excited	411.04 (80.76)	547.58 (109.68)	288.85 (70.20)	475.47 (107.22)	**–**
Sad	156.26 (32.57)	408.35 (86.82)	495.33 (127.75)	619.42 (148.20)	1 < 2,3**; 1 < 4***
Irritable	201.65 (45.55)	632.69 (145.64)	666.17 (186.09)	865.70 (224.45)	1 < 2,3**; 1 < 4***
Angry	110.64 (30.21)	368.31 (102.45)	553.23 (186.76)	830.72 (260.34)	1 < 2,3**; 1 < 4***

Key: *p ≤ .05, **p ≤ .01, ***p ≤ .001; Bold characters indicate findings NOT withstanding Bonferroni correction at *p* = .01; 1: Control group; 2: ADHD only group; 3: BPD only group; 4: Comorbid ADHD+BPD group

^a^ Means of squared successive difference of items used in the instability models

In models adjusted for anxiety and depression, the significant main effect of group in the instability of all items dissipated (*p* ≥ .09). (See section 6 in the supplementary materials for  main effects of group and Table S2 for adjusted estimated means of the instability models).

**Fig. 1:**
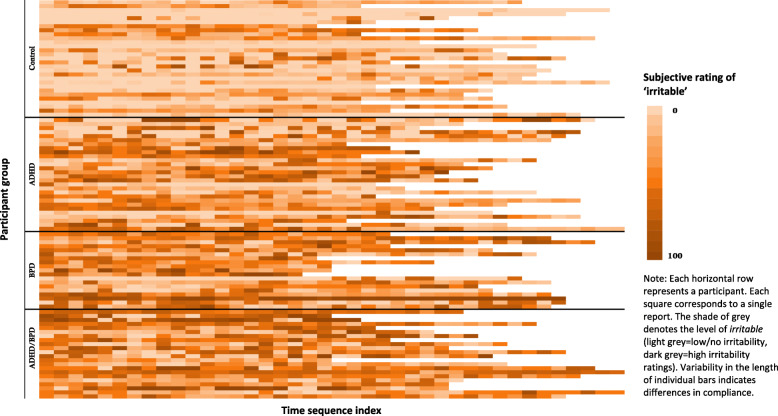
Heatmap of *irritable* ratings for the control and clinical groups

A heat map of the emotion ratings for *irritable* over the 5-day ambulatory monitoring period is shown in Fig. 1, illustrating the pattern of frequency, intensity and instability of emotional symptoms in the different groups.

### Impact of bad events on intensity of negative mood

There was a significant main effect of group on the frequency of reported bad events (*bad social *event: X^2^(3) = 16.61, *p* = .001; *bad functional *event: X^2^(3) = 12.90, *p* = .005). Pairwise comparisons indicated that *bad social* events were more frequent in the ADHD (*p* = .03), BPD (*p* = .003) and comorbid ADHD+BPD (*p* = .009) groups compared to controls, with no differences between the three clinical groups (*p* = 1). However, *bad functional* events were only more frequent in the ADHD group compared to the control group (*p* = .003), with all other group comparisons being non-significant, *ps* ≥ .17 (see Table 2 for means and SDs).

Multilevel models for intensity of *sad*, *irritable*, and *angry* were repeated after including bad events as predictors. Models indicated that across the whole sample, *bad social* and *bad functional* events had a significant main effect on the intensity of negative emotion items, predicting an overall higher intensity of *sad*, *irritable* and *angry* (all *p* < .001). Further models also indicated a significant main effect of *bad social* and *bad functional* events in the three negative emotion items for all four groups (for *bad social *events: all *p* < .001, for *bad functional events*: .006 ≤ *p* < .0001). All findings were robust to the Bonferroni adjusted *p* = .01. Therefore, group differences for all models (reported in the "Intensity"section under "real-time emotional changes" ) remained unchanged indicating that bad events predict overall greater intensity of *sad*, *irritable*, and *angry* but don’t fully account for the group differences.

Finally, to test group differences in the affective response to bad events, multilevel models were repeated including an interaction term for bad events with group. There was no significant interaction between diagnosis and *bad functional* events on the intensity of *sad* (F(3, 2881) = 1.75, *p* = .15) and *irritable* (F(3, 2900) = .36, *p* = .78). However, there was a significant diagnosis by *bad functional* events interaction for intensity of *angry* (F(3, 2891) = 4.82, *p* = .002). Findings indicated that the effect of *bad functional* events on intensity of *angry* was significantly higher in the BPD (*difference of estimate*: -19.29, SE: 5.17, *p* = .004) and comorbid ADHD+BPD (*difference of estimate*: -20.16, SE: 4.98, *p* = .001) groups compared to the ADHD group. Significant group differences were robust to the Bonferroni adjusted *p* = .01. For *bad social* events, models indicated no significant interaction with diagnosis on the intensity of *sad* (F(3; 2859) = 1.01. *p* = .39), *irritable* (F(3, 2872) = 2.47, *p* = .06) and *angry* (F(3, 2865) = .50, *p* = .68). Results were unchanged in models controlling for anxiety and depression symptoms, and we therefore only reported results from unadjusted models.

## Discussion

The main aim of this study was to assess whether ED substantially differs between ADHD and BPD. Using questionnaire measures of ED, individuals with ADHD and those with BPD did not differ from one another, but reported significantly increased ED compared to controls. High-frequency assessment over 5 days provided more fine-grained data. Using this approach, the BPD and comorbid ADHD+BPD groups displayed significant differences compared to controls, with less positive and more negative intensity of emotions, and more instability of negative emotions. In contrast, the ADHD group did not differ from controls in the intensity of positive emotion items, but showed heightened intensity of certain negative emotions (with differences present in *irritable* but not *sad* and *angry)*, and increased instability of negative emotions (*sad*, *irritable* and *angry)* compared to controls. No differences were detected in the instability of positive emotion items between clinical groups and controls. Overall, these findings suggest that intensity and instability of positive emotions are not able to distinguish between individuals with ADHD and controls, contrary to clear differences in the intensity of emotions between controls and BPD populations.

We detected differences in the intensity of emotions between individuals with ADHD and those with BPD. Those with BPD displayed greater intensity of *sad* and lesser intensity of *happy* than those with ADHD, but no differences were detected between the clinical groups on intensity of *angry* and *irritable*. Further, no differences in the instability of positive and negative emotion items were seen between the three clinical groups. Overall, we can conclude that dynamics of ED are similar in ADHD and BPD, and cannot distinguish the disorders. The lack of differentiation in the intensity and instability of negative affect in ADHD and BPD was in line with non-specific patterns of ED also reported in bulimia nervosa, PTSD, depression, anxiety disorders and bipolar disorder [26].

All the above-mentioned case-control differences, as well as differences between ADHD and BPD dissipated after adjusting for symptoms of depression and anxiety. Depression and anxiety were strongly associated with intensity and instability of emotions in both ADHD and BPD. Future studies should address the underlying mechanisms of depressive and anxious symptoms, and how they relate to ED in ADHD and BPD. (See Table S3 in section 7 of the supplementary materials for the relationship between BSI scores of depression and anxiety, and ESM and questionnaire measures of ED).

Regarding the potential association between greater daily adversity and ED [31, 32], our exploratory analyses showed that individuals from all three clinical groups reported similar high incidence of *bad social* events compared to controls, and the ADHD group reported a higher incidence of *bad functional* events compared to controls only. Although it is not clear whether these are true differences in adverse events in the clinical groups compared to controls or reflect differences in the perception of adverse events in daily life.

Both *bad social* and *bad functional* events had a significant effect on the intensity of negative emotions across the sample. We also found that covarying for bad events did not alter the significant group differences identified, suggesting that the increased reporting of bad events did not drive differences in the intensity of the emotional symptoms in  any of the conditions. This finding is consistent with the previous ESM report in adult males with ADHD [17], and major depressive disorder [53], which found that although reactions to external events contributed to emotional intensity, they did not fully explain the heightened levels of emotional intensity.

For most of the emotions examined, the type of event (social or functional) had the same effect for both the ADHD and BPD groups. The exception was that the BPD group reported higher intensity of *angry* in the presence of *bad functional* events compared to the ADHD only group. This is in line with ESM findings reported by Kockler and colleagues [54], where BPD patients exhibited anger more frequently than clinical (PTSD, bulimia nervosa) or healthy control groups in the study. However, this isolated finding was not expected, and given the exploratory nature of the analyses, it should therefore be treated with caution. Overall, the findings did not support the initial hypothesis of ED being triggered more frequently by *bad social* events in BPD, as opposed to *bad functional* events in ADHD. This could be due to the predetermined frequency schedule of the ESM data which may not have been adequate to investigate the effects of adverse life events on negative emotions.

This study was the first to compare ADHD and BPD on measures of ED using a prospective ESM approach. Despite using multiple measures of ED and including carefully selected and diagnosed clinical cases, the findings should be considered in light of several limitations.

The compliance rate of around 75% was low compared to previous BPD studies with rates around 90% and above [15, 19], yet was more closely in line with other studies in outpatients with schizophrenia: 69% [55], and adult men with ADHD: 64% [17]. Lower compliance could result in fewer extreme data points and potentially mask differences in emotional changes in ADHD and BPD. However, studies with higher compliance have typically employed a different sampling frequency and duration (e.g. ratings were only over 24 h in the study by Ebner-Priemer and colleagues [19]). In the study by Solhan and colleagues [15], remuneration was contingent on the number of reports subjects completed each day. However, this approach could be problematic in BPD populations due to difficulties in reward processing [56].

We recruited an all-female clinical sample, limiting confounding by sex differences. BPD is more common in women in clinical samples [57], as approximately 75% of BPD diagnoses are in females [3]. Indeed, during the pilot phase of the study, we found this to be the case and experienced a very low rates of male referrals. This meant that recruitment of a sex-matched sample within the restricted timeframe of this study was not deemed feasible and so we proceeded by restricting recruitment to female participants only. So, although this limits interpretation of findings to female populations, it has the advantage of eliminating any confounding by sex. Additionally, results from the comparisons between the ADHD group and controls were in keeping with findings of Skirrow and colleagues [17] in adult men with ADHD and without other comorbidities, who showed increased intensity and instability of negative emotions and no differences in the intensity and instability of positive emotions compared to controls. On the other hand, to the best of our knowledge, no studies of ESM have been conducted in males with BPD. Two ESM studies in BPD initially recruited six [24] and two [33] males into their samples, but had to exclude them from analyses as the number was not sufficient for the examination of sex differences. Future studies in males with BPD are now required to examine if the findings reported here replicate in males with BPD.

Care was taken to exclude individuals with current comorbid major depressive disorders and anxiety disorders. Yet, all three clinical groups showed high levels of anxiety and depression symptoms compared to controls, and significant differences between groups in measures of ED in ESM data were all accounted for by anxiety and depression symptoms. Additionally, eight ADHD cases, 15 BPD cases and eight comorbid ADHD+BPD cases were on concomitant anti-depressants for co-occurring mild depressive symptoms, which constituted around 45% of the total clinical sample (*n* = 31). Excluding participants on anti-depressants would have made our sample unrepresentative of the ADHD and BPD populations, as the high rate of 45% in this study shows. To run the main analyses without these cases, the clinical sample size would have greatly decreased (ADHD = 20, BPD = 4, ADHD+BPD = 14), making between group comparisons unmeaningful. Therefore, we could not run any sensitivity analyses with the exclusion of these cases.

Further, our findings are based on a clinical sample without current Axis I comorbidities, an important exclusion criterion to make sure the dynamics of ED were not affected by other (undiagnosed or uncontrolled) comorbidities, and attribute findings accurately to the disorders under investigation. This has nevertheless the limitation of our sample not being representative of ADHD and BPD populations, which often present with comorbidities.

Whether assessed using retrospective questionnaires or by ESM, all reports were based on the subjective view of the individuals of their affective states. Future investigations of ED in ADHD and BPD using ESM approach should consider incorporating more objective physiological measures (e.g. heart rate, breathing, arousal) that capture objective autonomic responses which can be associated with emotional changes [58].

Finally, although our findings indicate that ED is expressed to a similar degree of intensity and instability in ADHD and BPD, it’s worth acknowledging that this conclusion is based on two widely used markers of ED only, and future research could incorporate other markers such as cue reactivity or return to baseline.

## Conclusions

The current study characterised the intensity and instability in emotions experienced by adult females with ADHD and/or BPD over a period of five days. Findings supported the notion that ED has a valuable trans-diagnostic clinical pattern in both conditions and is unlikely to be helpful in distinguishing between ADHD and BPD. Further research should now explore overlapping mechanisms of ED using dimensions of neurobiology and observable behaviour, as suggested by the Research Domain Criteria [59] and use dimensional measurements of ADHD and BPD in population samples to replicate findings reported here.

## Supplementary Information

**Additional file 1.**

## Data Availability

The datasets used and/or analysed during the current study are available from the corresponding author on reasonable request.

## Abbreviations

ADHD: Attention Deficit Hyperactivity Disorder
ALS-SF: Affective Lability Scale- Short Form
BPD: Borderline Personality Disorder
BSI: Brief Symptom Inventory
DIVA: Diagnostic Interview for ADHD in Adults
ED: Emotional Dysregulation
ESM: Experience Sampling Method
IQ: Intelligence Quotient
PTSD: Post Traumatic Stress Disorder
SSD: Squared Successive Difference
WRAADDS-EDS: Wender-Reimherr Adult Attention Deficit Disorder Scale- Emotion dysregulation subscale
ZAN-BPD: Zanarini rating scale for Borderline Personality Disorder
