# Supplementary material for: Comparable emotional dynamics in women with ADHD and borderline personality disorder
Source: Borderline Personal Disord Emot Dysregul. 2021 Feb 12;8:6. doi: 10.1186/s40479-021-00144-y (PMC7879647; doi:10.1186/s40479-021-00144-y)
Supplement: Supplementary file 1 — Additional file 1. [file 40479_2021_144_MOESM1_ESM.docx]

# **Clinical research diagnosis**

Attention deficit/hyperactivity disorder (ADHD) was assessed using the Diagnostic Interview for ADHD in Adults (DIVA). The DIVA is a validated structured interview for the assessment of adult ADHD according to DSM-IV diagnostic criteria. It consists of 18 questions (nine relating to inattentive symptoms and nine to hyperactive/impulsive), scoring the presence/absence of DSM-IV symptoms during both childhood and adulthood (1). Symptom onset and chronicity was established before the age of 12 (by the presence of “several” symptoms, defined as three or more in the current study) and the presence of more than five symptoms of inattention or hyperactivity/impulsivity in adulthood (in accordance with DSM-5 criteria) (1, 2). Subjects met all other criteria for DSM-5 ADHD diagnosis including pervasive impairments from the symptoms in more than one setting.

Borderline Personality Disorder (BPD) diagnosis was established by the presence of five (or more) of the nine diagnostic symptoms for the disorder in accordance to the DSM-5 (2). BPD was assessed using the Zanarini rating scale for Borderline Personality Disorder (ZAN-BPD). The ZAN-BPD is a semi-structured interview that generates a continuous measure assessing borderline psychopathology (3). Each of the nine items is rated on a five-point anchored rating (0=no symptoms to 4=severe symptoms), generating a maximum score of 36. The ZAN-BPD has high internal consistency (Cronbach’s α=.85) and significant test-retest reliability (*p* < .001). The ZAN-BPD was used as a proxy for BPD diagnostic criteria: a symptom was marked as present if an item had a score of two or above, which is equivalent to being rated as ‘threshold or true’ on the Structured Clinical Interview for DSM-IV (SCID-II) BPD criteria (4). Therefore, a BPD research diagnosis was established when a participant had a score of two or above on at least five of the nine items on the ZAN-BPD.

Initial diagnoses were determined by clinicians, prior to being referred to the research study. To establish a research diagnosis, the DIVA and ZAN-BPD, as detailed above, were carried out by the lead researcher (TM), with no disagreement with the clinical diagnoses made previously. In few cases, research diagnoses were done by research assistants, that were subsequently discussed and reviewed with the lead researcher.

# **Alcohol and drug use**

Alcohol use in the last 12 months was assessed by the Alcohol Use Disorders Identification Test, an alcohol screen that can help identify individuals who are hazardous drinkers or have active alcohol use disorders (abuse or dependence). It is scored on a scale of 0 (no alcohol use) to 4 (daily or almost daily) (5). Frequent drug use, defined as use of recreational drugs **several times a week or more** in the last 12 months was assessed by a substance use checklist, a short screen developed by our research team to identify patterns of illegal drug use in the past 12 months measured on a scale of 0 (never used) to 5 (several times a day).

High risk of alcohol dependence on the AUDIT-C, was defined as having a total score of 20 or above (as per the screener’s guideline). Five participants from the clinical groups (1 ADHD, 2 BPD, 2 comorbid ADHD+BPD) reported elevated risk of alcohol dependence, and seven participants (1 control, 2 ADHD, 1 BPD, 3 comorbid ADHD+BPD) reported elevated risk of substance dependence (i.e. legal highs, opiates, cocaine, and cannabis). In addition, two clinical cases (1 BPD, 1 comorbid ADHD+BPD) reported both elevated risk of substance and alcohol dependence.

Sensitivity analyses were conducted on the main outcomes of emotional dysregulation (ED) measured by experience sampling method (ESM) and retrospective rating scales after removing the above-mentioned 14 participants. Findings from the ESM data were not altered by excluding these individuals, and we can therefore assume ESM results were not driven by these cases. Regarding retrospective self-report measures of ED (Affective Lability Scale-Short Form (ALS-SF) and Wender-Reimher Adult Attention Deficit Disorder Scale- Emotional dysregulation subscale (WRAADDS-EDS), findings revealed that in addition to the significant case-control differences (similar to findings for the tests without excluding the 14 individuals), all three clinical groups also significantly differed from one another (*p* < .001) on both scales, with the ADHD group reporting lower scores compared to the BPD group, who also reported lower scores than the comorbid ADHD+BPD group.

# **Symptom measures**

The ALS-SF (6) is comprised of 18 items scored 0-3 (very un-descriptive, rather un-descriptive, rather descriptive, very descriptive), and measures swift fluctuations from normal (euthymic) mood to elation, depression, and anger. Total overall score of the ALS was used as an outcome variable.

The WRAADDS-EDS assesses temper, affective lability and emotional over-reactivity and shows high internal consistency (Cronbach’s α= .78) and good test-retest reliability (r= .96) (7). Total overall score of the emotional dysregulation subscale of the WRAADDS was used as an outcome variable.

Co-occurring depression and anxiety were measured by subscales of the Brief Symptom Inventory (BSI) (8). The BSI is a self-rated measure consisting of 53-items evaluating psychological distress and psychiatric disorders in nine domains including depression and anxiety on a 4-point Likert-scale (0=not at all to 3=extremely). The BSI has good internal reliability of .7 and robust test-retest reliability of .68 (8). The BSI depression and anxiety subscales showed good predictive validity for DSM-IV depression (area under the curve (AUC) = .89, *p* <.001) and anxiety (AUC = .80, *p* < .001) (9).

# **Pre-Processing of ESM Data**

In intensive longitudinal data, there are clustering issues to take into account (10), because observations from the same clusters are usually more identical than observations from different clusters. Mixed models take into account the assumption of independence of observations, and give an estimate of the correlations in the same cluster (10). When there is consistency among a cluster’s responses, then there is variation among the clusters’ means: the between-cluster variance. The ratio of the between-cluster variance to the total variance (the sum of between-cluster and within-cluster variability) is called the intra-class correlation (ICC) (11). We calculated ICC for all outcome variables, and found it to be between 30-80%, which is the acceptable range (10, 12).

# **Intensity models adjusted for depression and anxiety**

Main effect of group

Happy: F (3, 92.37) = .55, *p* = .65

Excited: F (3, 92.29) = .50, *p* = .68

Sad: F (3, 92.42) = 1.03, *p* = .38

Angry: F (3, 92.32) = .35, *p* = .79

**Table S1** Estimated means and standard errors from intensity models adjusted for depression and anxiety

|  | Estimated Mean (Standard Error) | | | | Post-hoc |
| --- | --- | --- | --- | --- | --- |
|  | Control^1^ | ADHD^2^ | BPD^3^ | ADHD+BPD^4^ |  |
| Happy | 48.58 (3.63) | 48.94 (2.81) | 42.82 (3.72) | 46.39 (4.10) | - |
| Excited | 37.61 (4.27) | 36.18 (3.31) | 30.74 (4.38) | 35.76 (4.83) | - |
| Sad | 29.84 (3.90) | 31.85 (3.02) | 33.95 (4.00) | 25.99 (4.40) | - |
| Irritable | 24.91 (3.88) | 37.60 (3.00) | 39.82 (3.98) | 39.33 (4.38) | **1 < 2*** |
| Angry | 21.10 (3.87) | 22.99 (3.00) | 26.47 (3.97) | 21.10 (3.87) | - |

Key: **p*≤.05; Bold characters indicate findings not withstanding Bonferroni correction at *p* = .01.

# **Instability models adjusted for depression and anxiety**

Happy: F (3, 89.35) = .68, *p* = .56

Excited: F (3, 93.44) = .89, *p* = .45

Sad: F (3, 91.78) = 1.57, *p* = .20

Irritable: F (3, 90.84) = 2.21, *p* = .09

Angry: F (3, 92.90) = .88, *p* = .45

**Table S2** Estimated means and standard errors from instability models adjusted for depression and anxiety

|  | Estimated Mean (Standard Error) | | | |
| --- | --- | --- | --- | --- |
|  | Control^1^ | ADHD^2^ | BPD^3^ | ADHD+BPD^4^ |
| Happy | 299.41 (65.11) | 386.58 (65.23) | 304.82 (67.82) | 409.85 (100.37) |
| Excited | 373.62 (101.27) | 487.80 (102.49) | 336.04 (93.24) | 545.38 (166.56) |
| Sad | 241.37 (68.39) | 483.89 (106.35) | 346.89 (100.58) | 376.64 (120.19) |
| Irritable | 266.12 (83.84) | 686.82 (167.67) | 542.77 (175.10) | 633.29 (224.90) |
| Angry | 226.94 (83.44) | 449.43 (128.02) | 330.18 (124.33) | 374.00 (155.03) |

# **Relationship between BSI scores of depression and anxiety and measures of emotion instability**

Given the substantial effects of depression and anxiety symptoms in the intensity and instability models, Table S3 below shows the correlations between BSI scores of depression and anxiety, and retrospective and ESM measures of ED. SSD scores were aggregated over the five-day assessment period to calculate the mean SSD (MSSD) for each item that we subsequently used in the cross-validation analyses (13).

**Table S3** Correlation coefficients between MSSDs for ESM items and retrospective questionnaire measures

| MSSD | BSI_depression | BSI_anxiety | WRAADDS-EDS | ALS |
| --- | --- | --- | --- | --- |
|  |  |  |  |  |
| Happy | **.28** | **.29** | **.33** | **.36** |
| Excited | .02 | .08 | .17 | .18 |
| Sad | **.40** | **.41** | **.37** | **.37** |
| Irritable | **.33** | **.37** | **.33** | **.30** |
| Angry | **.52** | **.57** | **.50** | **.46** |

Key: Correlations in bold are significant at the .01 level (2-tailed).

Apart from mean instability of *excited*, mean SSDs show moderate to strong correlations with BSI scores of anxiety and depression, as well as with retrospectively assessed questionnaire measures.

References

1. Kooij SJJ. Adult ADHD. 3 ed: Spring-Verlag London; 2013. XVII, 294 p.

2. American Psychiatric Association. Diagnostic and Statistical Manual of Mental Disorders. 5th ed. Arlington, VA: American Psychiatric Publishing; 2013.

3. Zanarini M. Zanarini rating scale for Borderline Personality Disorder (ZAN-BPD): A continuous measure of DSM-IV borderline psychopathology. Journal of personality disorders. 2003;17(3):233-42.

4. First MB, Gibbon M, Spitzer RL, Williams JBW, Benjamin LS. Structured Clinical Interview for DSM-IV Axis II Personality Disorders, (SCID-II). Washington, D.C: American Psychiatric Press, Inc.; 1997.

5. Bohn MJ, Babor TF, Kranzler HR. The Alcohol Use Disorders Identification Test (AUDIT): validation of a screening instrument for use in medical settings. J Stud Alcohol. 1995;56(4):423-32.

6. Oliver MNI, Simons JS. The affective lability scales: Development of a short-form measure. Personality and Individual Differences. 2004;37(6):1279-88.

7. Wender PH. Attention-deficit hyperactivity disorder in adults. New York: Oxford University Press; 1995.

8. Derogatis LR. BSI Brief Symptom Inventory: Administration, Scoring, and Procedure Manual. 4th Ed. ed. Minneapolis, MN: National Computer Systems; 1993.

9. Petkus AJ, Gum AM, Small B, Malcarne VL, Stein MB, Wetherell JL. Evaluation of the factor structure and psychometric properties of the brief symptom inventory—18 with homebound older adults. International Journal of Geriatric Psychiatry $V 25. 2010(6):578-87.

10. Aarts E, Verhage M, Veenvliet JV, Dolan CV, van der Sluis S. A solution to dependency: Using multilevel analysis to accommodate nested data. Nature Neuroscience. 2014;17:491.

11. Heck RH, Thomas SL. An introduction to multilevel modeling techniques: MLM and SEM approaches using Mplus. Third ed. New York and London: Routledge; 2015.

12. Bolger N, Laurenceau J-P. Intensive Longitudinal Methods: An introduction to diary and experience sampling research. Little TD, editor. New York: Guilford Press; 2013.

13. Solhan MB, Trull TJ, Jahng S, Wood PK. Clinical assessment of affective instability: Comparing EMA indices, questionnaire reports, and retrospective recall. Psychological assessment. 2009;21(3):425-36.
